# Sepsis following hysteroscopic removal of retained products of conception: A case report and literature review

**DOI:** 10.1097/MD.0000000000044391

**Published:** 2025-09-05

**Authors:** Yifei Dai, Linling Zhu, Fen Wang, Hao Chen, Xinyun Yang, Dingheng Li, Lei Chen

**Affiliations:** a Department of Gynecology, Hangzhou Women’s Hospital, Hangzhou, Zhejiang Province, P. R. China; b Department of Gynecology, Xiaoshan District Jingjiang Subdistrict Community Health Service Center, Hangzhou, Zhejiang Province, P. R. China; c Department of Pathology, Hangzhou Women’s Hospital, Hangzhou, Zhejiang Province, P. R. China; d Department of Reproductive Endocrinology, Women’s Hospital, Zhejiang University School of Medicine, Hangzhou, Zhejiang Province, P. R. China; e Department of Ultrasound, Hangzhou Women’s Hospital, Hangzhou, Zhejiang Province, P. R. China.

**Keywords:** antibiotic, complications, hysteroscopy, retained products of conception, sepsis

## Abstract

**Rationale:**

Sepsis following hysteroscopy is an rare complication, with current evidence suggesting that routine prophylactic antibiotic administration may not be warranted. However, this does not imply that we should disregard vigilance regarding the potential occurrence of severe infections post-hysteroscopy.

**Patient concerns:**

A 27-year-old female underwent hysteroscopic resection of retained products of conception after incomplete medical abortion. After 10 hours postoperatively, the patient developed hyperthermia (39.9°C), tachycardia (137 beats/min), and hypotension (77/36 mm Hg). The blood test results revealed elevations in procalcitonin to 15.02 ng/mL, leukocytosis count to 18.1 × 10^9^/L, neutrophils to 97.1%, and C-reactive protein level of 51.50 mg/L.

**Diagnoses:**

Sepsis was diagnosed according to the Quick Sepsis-related Organ Failure Assessment. Histopathological examination of retained products of conception demonstrated abundant inflammatory infiltrates, with immunohistochemical analysis revealing CD138-positive plasma cell clusters.

**Interventions:**

Empirical broad-spectrum antibiotics and fluid resuscitation were administered.

**Outcomes:**

The patient made a satisfactory recovery and was discharged 7 days postoperatively.

**Lessons:**

A retrospective analysis of post-hysteroscopic sepsis and septic shock cases was performed. Although postoperative infections exhibited a low incidence, our findings emphasize the necessity for increased clinical vigilance and prompt initiation of pathogen-directed therapies when severe infections arise.

## 1. Introduction

Retained products of conception (RPOC) refer to the clinical condition wherein placental and/or fetal tissue remains within the uterus following delivery, miscarriage, or termination of pregnancy. The presence of residual pregnancy tissue can lead to serious complications such as infection, postpartum hemorrhage, and intrauterine adhesions (IUA). Therefore, removal of retained products is essential for patients exhibiting significant bleeding or those suspected of having endometritis.

In recent years, the surgical management of RPOC has transitioned from blind curettage to visually guided procedures utilizing hysteroscopy. This advancement allows for the targeted removal of remnants, thereby minimizing damage to surrounding tissues and reducing the risk of IUA.^[1]^ Compared to traditional curettage, hysteroscopy demonstrates lower complication rates and improved subsequent pregnancy outcomes.^[2]^ Most studies report a low incidence of postoperative infections following hysteroscopic surgery, with rates ranging from 0.06% to 11.4%, generally negating the necessity for routine prophylactic antibiotics.^[3,4]^ Nevertheless, this study documents a case of sepsis following hysteroscopic removal of retained products, underscoring the critical need for vigilance regarding the potential for severe infections post-procedure. In addition, we conducted a literature review on serious infections following hysteroscopy to raise awareness among surgeons and to emphasize the importance of rigorous postoperative monitoring.

## 2. Case presentation

A 27-year-old gravida 3 para 0 woman with 2 prior induced abortions presented with a missed abortion. She underwent medical termination using 200 mg oral mifepristone and 600 μg oral misoprostol, resulting in embryonic tissue expulsion. Subsequent ultrasonography revealed an irregular intrauterine mass measuring approximately 4.0 × 3.5 × 2.2 cm, with detectable blood flow signals. Despite a recommendation for curettage, the patient opted for conservative management.

Following discharge, the patient experienced persistent minor vaginal bleeding, though she reported no celialgia or fever. Eleven days later, repeat ultrasonography showed a reduced intrauterine mass of approximately 3.2 × 2.5 × 1.7 cm, still exhibiting blood flow signals, serum beta-human chorionic gonadotropin levels were 474.6 IU/L. Clinical examination revealed no pelvic tenderness, and laboratory investigations – including complete blood count, C-reactive protein (CRP), leucorrhea routine screening, and liver and renal function tests – were normal. Tests for Chlamydia trachomatis and Neisseria gonorrhoeae were negative. Subsequently, hysteroscopic evacuation was performed to remove the RPOC. During the procedure, a mass measuring 4.0 × 2.0 × 2.0 cm was found adhering to the right uterine wall. The RPOC were systematically grasped and removed using ovum forceps and hysteroscopic 5 Fr toothed grasping forceps, ensuring the uterine cavity was cleared of all remnants. The operation lasted 35 minutes, during which the intraoperative blood loss was 50 mL.

**Figure 1.**
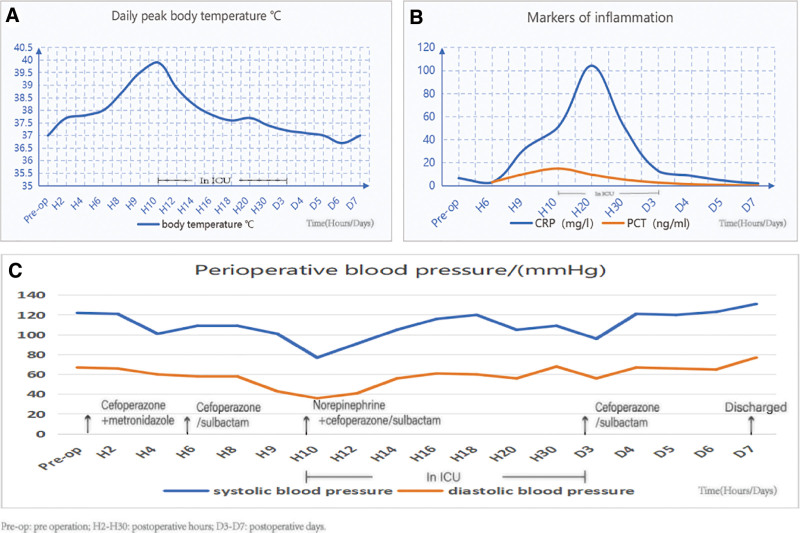
Line chart illustrating patient’s vital signs and inflammation markers during hospitalization. (A) Temperature during hospitalization. (B) Inflammation makers during hospitalization. (C) Perioperative changes in systolic blood pressure (SBP) and diastolic pressure (DBP). *D3–D7 = postoperation days, DBP = diastolic pressure, Pre-op = preoperative, H1–H30 = the first 30 postoperative hours, SBP = systolic blood pressure.

Postoperatively, the patient was administered prophylactic antibiotics (cefuroxime 1.5 g and metronidazole 0.5 g intravenously every 12 hours) to prevent infection. At 6.5 hours postoperatively, the patient developed fever (38°C) and tachycardia (108 beats/min), with a Quick Sepsis-related Organ Failure Assessment (qSOFA) score of 0. Laboratory results showed: white blood cell count 8.4 × 10⁹/L (94.2% neutrophils), hemoglobin 122 g/L, CRP 3.09 mg/L, and procalcitonin (PCT) 3.19 ng/mL. Antibiotics were escalated to intravenous cefoperazone/sulbactam (2 g every 8 hours). By 10 hours postoperatively, clinical deterioration ensued with hyperpyrexia (39.9°C), marked tachycardia (137 beats/min), and hypotension (77/36 mm Hg), with a qSOFA score of 2. Laboratory findings demonstrated: leukocytosis (white blood cell count 18.1 × 10⁹/L; 97.1% neutrophils), elevated CRP (51.50 mg/L), markedly elevated PCT (15.02 ng/mL; showed in Fig. 1).

**Figure 2.**
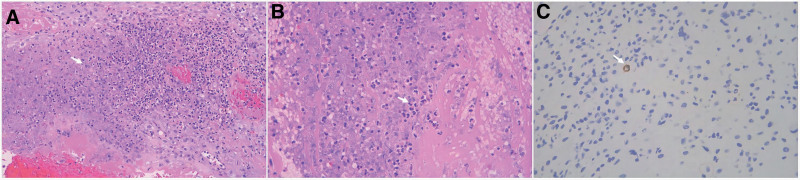
RPOC with endometritis. (A) Acute infection necrosis (white arrow) in RPOC with hematoxylin and eosin (H&E), magnification (200×). (B) Neutrophils (white arrow) in necrotic area with H&E, magnification (400×). (C) Immunohistochemical staining for CD138, presenting the positivity with white arrow, magnification (400×). H&E = hematoxylin and eosin, RPOC = retained products of conception.

Consequently, the patient was transferred to the intensive care unit for fluid resuscitation and continuous electrocardiograph monitoring. Additionally, the intensive care unit team initiated a combination antibiotic regimen of cefoperazone-sulbactam and linezolid (600 mg every 12 hours). Diagnostic imaging, including pelvic magnetic resonance imaging and chest X-ray, revealed no abnormalities. After treatment, the patient’s condition was gradually relieved, which was characterized by stable recovery of vital signs, gradual decline of infection indicators. Blood, urine, and cervical secretions cultures returned negative. Consequently, linezolid was discontinues and the patient returned to Gynecology ward on postoperative day 3. Pathological examination of the RPOC revealed degenerated chorionic villi and decidua with significant neutrophils infiltration and CD138-positive plasma cells (showed in Fig. 2). The cefoperazone/sulbactam regimen was continued for a total of 7 days. The patient recovered uneventfully and was discharged 7 days postoperatively. Furthermore, serum hCG levels returned to baseline within 21 days postoperatively.

Patient Perspectives: Following recovery, the patient described the onset of sepsis episode as “sudden and terrifying,” and expressed persistent apprehension toward future hysteroscopic procedures.

## 3. Cases review and methods

This study was conducted in accordance with the Declaration of Helsinki and approved by the Hangzhou Women’s Hospital ethics committee (2024 A No. 027). Written informed consent was obtained from the patient for publication of this case report and accompanying images.

To systematically identify risk factors for post-hysteroscopic infection, we conducted a comprehensive literature review. All relevant English-language literature was searched in the PubMed, Web of Science, and EMBASE databases from 1968 to July 2024. The search utilized combinations of Medical subject heading terms, key words, and word variants of “sepsis,” “septic shock,” “shock,” “hysteroscopy,” “hysteroscope,” and “hysteroscopic surgery.” The inclusion criteria for this review encompassed randomized clinical trials and non-randomized controlled studies (including observational prospective and retrospective cohort studies, case-control studies, and case series), with a focus on studies that diagnosed sepsis or shock subsequent to hysteroscopic surgery. Additional pertinent reports were identified through a manual search of the reference lists of relevant articles and reviews. Exclusion criteria were as follows: studies for which the full text could not be obtained, studies with overlapping information from other publications, and meta-analyses.

Following a comprehensive review of all identified cases and the available English literature, a total of 9 cases were included in our analysis.^[2,5–11]^ The clinical data for each case are presented in Table 1. The age at diagnosis of these cases ranged from 23 to 68 years old. The cohort comprised 2 postmenopausal women, 6 premenopausal women, and 1 patient with unknown menopausal status. Procedural indications included diagnostic hysteroscopy (n = 2) and therapeutic hysteroscopy (n = 6). The therapeutic procedures consisted of endometrial polypectomy (n = 2), submucosal fibroid resection (n = 2), endometrial ablation (n = 1), and removal of RPOC (n = 1); specific details for 1 case were unavailable. Preoperative evaluations revealed no signs of infection, and prophylactic antibiotic use was not consistently reported. Pathogens isolated from blood cultures included *Escherichia coli* (n = 2), *Streptococcus species* (n = 2), *Klebsiella pneumoniae* (n = 1), and *Staphylococcus aureus* (n = 1); blood cultures were negative in three patients. Imaging revealed pelvic abscess formation in 3 patients; the remaining patients showed no evidence of uterine perforation or pelvic/abdominal abscess. Two of the 9 reported cases were fatal.

**Table 1:** Overview of published cases of sepsis following hysteroscopy.

Author	Age of patient (yr)	Disease	Operation	Symptom onset	Primary symptoms	Identified pathogen	Antibiotics	Prognosis	Comments
Parkin DE^[5]^	40	Uterine bleeding	Hysteroscopic endometrium excision	1 d	Fever, watery diarrhea, toxic shock syndrome	*Staphylococcus aureus* (blood and peritoneal)	Intravenous (IV) cefotaxime + metronidazole + gentamicin	MODS, death	Hypodermic goserelin 3.6 mg at 5 wk before surgery
Bhagat N^[6]^	68	Endometrial hyperplasia	Hysteroscopy	15 h	Fever, celialgia, vomit, toxic shock syndrome	Beta-hemolytic Group A *Streptococci* (blood and vaginal)	IV augmentin + metronidazole/Gentamicin (Days 1–3); IV benzylpenicillin + clindamycin (Days 4–8)	Recovery	–
Golan A^[2]^	Not mentioned	RPOC	Hysteroscopic removal of RPOC	Not mentioned	Sepsis, diffuse intravascular coagulopathy	Not mentioned	Not mentioned	Recovery	Delivered 7 d before surgery
Kobayashi Y^[7]^	Not mentioned	Not mentioned	Not mentioned	Not mentioned	Abdominal abscess, septic shock	*Klebsiella, pneumoniae* (abscess)	Not mentioned	Not mentioned	Use of immunosuppressants after liver transplantation
Meneses T^[8]^	39	Uterine submucous myoma	Hysteroscopy	5 d	Fever, celialgia, right fallopian tube abscess, septic shock	Blood culture negative	IV large-spectrum antibiotics	MODS, hysterectomy with bilateral salpingo-oophorectomy	Hysteroscopy procedure discontinued due to excessive bleeding a month ago
Belouad M^[9]^	60	Endometrial polyp	Endometrial polypectomy + endometrial biopsy	48 h	Fever, diffuse abdominal pain, pyometrium	*E coli* (blood)	IV imipinem with amikacine	Death	–
Chua KJC^[10]^	50	Uterine submucous myoma	Electrotomy of uterine submucous myoma	1 d	Fever, septic shock	Group B *streptococcus* (blood)	IV gentamicin + clindamyci + vancomycin	Recovery	Uterine artery embolization 15 mo ago
Chua KJC^[10]^	43	Uterine submucous myoma	Electrotomy of uterine submucous myoma	2 d	Fever, septic shock	*Coli* (Uterine secretion and urine)	IV piperacillin/tazobactam (Day 1); IV piperacillin/tazobactam (Days 2–7)	Hysterectomy	Preoperative anemia
Su D^[11]^	23	Endometrial polyp	Endometrial polypectomy	3 h	Fever, celialgia, septic shock	Blood culture negative	IV piperacillin tazobactam + tinidazole	Recovery	History of recurrent vaginitis

IV = intravenous, MODS = multiple organ dysfunction syndrome.

## 4. Discussion

### 4.1. Case-based discussion

RPOC represent a common complication following childbirth or miscarriage. In situations where expectant management or medical treatment is inadequate, surgical intervention becomes necessary. One significant risk associated with such interventions is the development of IUA, which typically result from endometrial trauma caused by procedures such as dilation and curettage, cesarean sections, uterine surgeries, or infections.^[12]^ These adhesions may lead to menstrual disturbances and infertility, and pregnancies may be complicated by miscarriage, ectopic pregnancy, abnormal placentation, fetal growth restriction, fetal anomalies, premature labor and delivery, and postpartum hemorrhage.^[13]^

Hysteroscopy allows for direct visualization of the uterine cavity, facilitating the precise removal of RPOC while preserving endometrial tissue. This targeted approach minimizes endometrial damage and the risk of IUA, thereby improving future reproductive outcomes.^[1]^ Its minimally invasive nature makes it increasingly popular for treating intrauterine RPOC. However, the incidence of infection following hysteroscopic surgery for RPOC remains uncertain. Most studies suggest that hysteroscopic procedures, whether diagnostic or therapeutic, are associated with a low infection rate.^[3,14]^ Nonetheless, a retrospective analysis of cases presenting with sepsis or septic shock following hysteroscopy identified 9 cases of severe sepsis, 2 of which were fatal. These findings highlight the necessity for ongoing vigilance in monitoring complications associated with hysteroscopy.

We report a novel case of sepsis following hysteroscopic removal of RPOC, supplementing existing literature on this complication. Pathological examination in our case revealed abundant inflammatory infiltrates with CD138-positive plasma cells within the RPOC, demonstrating that RPOC can trigger endometrial plasma cell recruitment, thereby precipitating endometritis. The pathogenesis of postoperative sepsis likely involved hysteroscopic distension pressure facilitating inflammatory cell translocation into the bloodstream. Notably, 2 modifiable risk factors were identified in our case: Large RPOC diameter: Consistent with prior research, larger residual tissue volume correlates with higher post-hysteroscopic complication rates.^[15]^ Prolonged operative duration: Extended surgical time may exacerbate bacterial dissemination. Furthermore, staged hysteroscopic RPOC removal has been reported to increase the risk of postoperative fever.^[16]^ These findings position RPOC – particularly large-volume remnants – as a significant risk factor for post-hysteroscopic sepsis. However, the current evidence remains observational; larger cohort studies are needed to validate the association between RPOC characteristics (size, operative time) and infection risk. Additionally, our results emphasize that the indications for hysteroscopic RPOC removal should be strictly controlled.

### 4.2. Literature review and synthesis

To systematically identify risk factors for post-hysteroscopic infection, we conducted a comprehensive literature review that expanded upon our initial case observations. Our analysis revealed 9 documented cases of severe infection, with 3 occurring after hysteroscopic myomectomy and 2 following polypectomy. This observation suggests that uterine fibroids^[17]^ and endometrial polyps^[18]^ may represent additional risk factors, likely through their established association with chronic endometritis. These findings complement our earlier report of sepsis following RPOC removal, collectively indicating that various intrauterine pathologies may predispose to infection through shared inflammatory mechanisms.

A large-scale study of 42,934 hysteroscopy cases further demonstrated elevated infection risk in patients with ovarian endometriosis and adenomyosis.^[3]^ Notably, all 5 patients with these conditions who developed postoperative adnexal inflammatory masses had either endometriosis or adenomyosis. The pathogenic mechanism may involve: translocation of pathogens or inflammatory mediators from chronic hemorrhagic endometriotic lesions that provide a favorable microenvironment for bacterial proliferation, and the distension medium used during the procedure might facilitate the introduction of endometrial cells or vaginal and cervical flora into the peritoneal cavity via the fallopian tubes.^[3]^ The generated intrauterine pressure may further promote vascular dissemination of inflammatory components, potentially explaining both localized inflammatory responses and systemic infections.

In conclusion, although severe infections remain rare, clinicians must maintain heightened vigilance following therapeutic hysteroscopy. Current evidence does not support the routine use of prophylactic antibiotics for hysteroscopic surgery, with the majority of literature indicating no reduction in postoperative infection rates.^[4]^ However, based on our synthesis of identified infection risk factors, we suggest considering prophylactic antibiotics for patients with specific high-risk features during hysteroscopy. These factors include RPOC, submucosal myoma, endometriosis and adenomyosis. Prophylaxis decisions for these subgroups should be individualized based on clinical judgment pending further evidence. Importantly, clinicians must recognize that postoperative infections may present atypically. Our literature review indicates that postoperative infection can manifest as isolated fever, even when routine imaging and blood culture examinations yield negative results. In such scenarios, sepsis should be suspected if postoperative fever coincides with elevated PCT levels. Clinicians should perform risk stratification according to sepsis guidelines, using qSOFA for early detection. Upon detection of systemic infection, microbiological cultures (e.g., blood, urine) should be collected promptly,^[19]^ and continuous electrocardiography and pulse oximetry monitoring should be initiated. Patients with septic shock or multiple organ failure require emergent intensive care admission, with immediate resuscitation measures (intravenous fluids and broad-spectrum antibiotics).

Several limitations warrant acknowledgments: Our literature review primarily identified English-language case reports, introducing potential selection bias and precluding meta-analysis; The absence of prospective comparative data limits definitive conclusions regarding risk stratification. Future prospective cohort studies are need to quantify infection risks associated with specific pathologies (e.g., submucosal fibroids, RPOC, endometriosis, and adenomyosis). Concurrent laboratory investigations should elucidate underlying mechanisms, including roles of altered endometrial receptivity, disrupted mucosal barriers, and distension pressure-mediated microbial or cellular translocation. Additionally, future research should explore novel prognostic interventions, such as Vitamin D supplementation, which may potentially enhance pathogen clearance,^[20]^ and reduce disease severity.^[21]^

## Author contributions

**Conceptualization:** Xinyun Yang.

**Data curation:** Yifei Dai, Linling Zhu, Hao Chen.

**Formal analysis:** Yifei Dai, Linling Zhu.

**Funding acquisition:** Yifei Dai, Linling Zhu, Xinyun Yang.

**Investigation:** Fen Wang, Dingheng Li.

**Resources:** Hao Chen.

**Supervision:** Lei Chen.

**Visualization:** Hao Chen.

**Writing – original draft:** Yifei Dai, Linling Zhu.

**Writing – review & editing:** Lei Chen.
